# TMED3 promotes prostate cancer via FOXO1a and FOXO3a phosphorylation

**DOI:** 10.32604/or.2024.048054

**Published:** 2024-12-20

**Authors:** XIUWANG WEI, JIANBO LIANG, HUANWEN HUANG, DAMING YANG, XINXIN WANG, XIUJIA WANG, CHANGSHENG CHEN, KAIQIANG LI, TAISEN PANG, BIN HU, FENGNING WU

**Affiliations:** 1 Department of Urology, The People’s Hospital of Guangxi Zhuang Autonomous Region, Nanning, 530000, China; 2 Department of Rehabilitation, The People’s Hospital of Guangxi Zhuang Autonomous Region, Nanning, 530000, China

**Keywords:** Prostate cancer, Transmembrane emp24 trafficking protein 3 (TMED3), forkhead box O transcription factor (FOXO), Proliferation, Apoptosis

## Abstract

**Background:**

Transmembrane emp24 trafficking protein 3 (TMED3) is associated with the development of several tumors; however, whether TMED3 regulates the progression of prostate cancer remains unclear.

**Materials and Methods:**

Short hairpin RNA was performed to repress TMED3 in prostate cancer cells (DU145 cells) and in a prostate cancer mice model to determine its function in prostate cancer *in vitro* and *in vivo*.

**Results:**

In the present study, we found that TMED3 was highly expressed in prostate cancer cells. *In vitro*, shTMED3 treatment suppressed the proliferation, invasion, and migration and promoted the apoptosis of DU145 cells. Additionally, the Kyoto Encyclopedia of Genes and Genomes pathway enrichment analysis showed a strong correlation between TMED3 and forkhead box O transcription factor (FOXO) pathway. Furthermore, TMED3 inhibition efficiently decreased FOXO1a and FOXO3a phosphorylation. *In vivo*, TMED3 downregulation suppressed the apoptosis, growth, and metastasis of prostate cancer cells via FOXO1a and FOXO3a.

**Conclusion:**

The present findings show that TMED3 participates in the regulation of prostate cancer progression via FOXO1a and FOXO3a phosphorylation, thereby revealing a novel mechanism underlying prostate cancer development and suggesting that TMED3 inhibition may serve as a novel strategy for prostate cancer treatment.

## Introduction

Prostate cancer, the most commonly diagnosed cancer in men, is an androgen-dependent tumor. Androgen can stimulate prostate cancer cell growth and disease progression. Despite many advances in traditional therapies including androgen deprivation therapy and antiandrogen therapy may be useful for early-stage prostate cancer [1–4]. However, the therapeutic effect of such approaches on prostate cancer is limited once the cancer cells metastasize to distant organs. Thus, the elucidation of molecular mechanisms underlying prostate cancer development and progression will help improve treatment strategies against the cancer.

Prostate cancer exhibits characteristics of increased proliferation, migration, and invasion and decreased apoptosis of prostate cancer cells [5–7]. Transmembrane emp24 domain (TMED)-containing proteins have been recognized for their role in pancreatic diseases, acute inflammatory responses, and cancer [8–10]. Emerging evidence has shown that TMED3 is closely related to cancer tumorigenesis progression, proliferation, invasion, and metastasis [11,12]. High-throughput transcriptomics and RNAi analysis in a previous study showed that the mRNA expression of ERGIC1 and TMED3 was upregulated in cultured prostate cancer cells [13]. However, the involvement of TMED3 in the pathophysiological progression of prostate cancer cells and the potential molecular mechanisms remain unclear.

The forkhead box O transcription factor (FOXO) family has recently been highlighted as crucial for diseases [14,15]. FOXO1a and FOXO3a activation plays a protective role in prostate cancer [16,17]. Furthermore, FOXO1a phosphorylation and inhibition contribute to cell proliferation and survival in prostate cancer [16]. Additionally, FOXO3a inhibition enhances the migration of prostate cancer cells and differential regulation of epithelial–mesenchymal transition (EMT) markers [18]. Similarly, blocking FoxO3a activity accelerates prostate cancer progression *in vivo* [19]. Thus, in the present study, we hypothesize that the TMED3-induced phosphorylation of FOXO1a and FOXO3a may modulate the proliferation, migration, invasion, and apoptosis of prostate cancer cells, thereby causing prostate cancer.

Herein, initially, we examined the proliferation, migration, invasion, and apoptosis of prostate cancer cells after knocking down TMED3 *in vitro*. Next, we investigated the possible downstream signaling pathway of TMED3 using gene chips and analyzed the causative association of TMED3 and the FOXO pathway by performing the Kyoto Encyclopedia of Genes and Genomes (KEGG) pathway enrichment analysis. Finally, we determined whether TMED3 downregulation alleviated prostate cancer development *in vivo*. The present results suggest that TMED3 serves as a potential tumor-promoting gene that enhances the proliferation and metastasis of prostate cancer, and TMED3 inhibition may be a useful therapeutic target against prostate cancer.

## Materials and Methods

### Chemicals and reagents

An apoptosis kit (88–8007) was purchased from eBioscience (CA, USA). The following antibodies were purchased from Abcam (Cambridge, MA, USA): anti-TMED3 (ab223175), anti-phosphorylation FOXO1a (ab131339), anti-phosphorylation FOXO3a (ab154786), anti-laminB (ab133741), anti-Ki67 (ab16667), and anti-GAPDH (ab8245). The CCK8 kit (96992) and antibody-propidiumiodide (PI) (P4170) were obtained from Sigma-Aldrich (St. Louis, USA). Hematoxylin and eosin were obtained from Solarbio Science & Technology (Beijing, China).

### In vitro experiments

The human prostate cancer cell line DU145 without mycoplasma was obtained from Procell Life Science & Technology Co., Ltd. DU145 cells were cultured using DMEM with 10% FBS, penicillin and streptomycin under 5% CO_2_ at 37 °C. To silence TMED3 in DU145, the cells were seeded in 6-well plates and infected with a lentivirus containing TMED3 short hairpin RNA (shRNA) (shTMED3 group) target sequences. A negative control (shCtrl group) was also set. The cells were then cultured at 37°C for 72 h. The recombinant lentivirus plasmid targeting shTMED3-1 (CTCTCACAAGACCGTCTACTT), shTMED3-2 (CACCTTCGAGCTGCCGGACA-A), shTMED3-3 (CGTGAAGTTCTCCCTGGATTA), and the control shRNA (ACGACGTCAGCTGGTGCATGT) were developed.

### Animal treatment and in vivo imaging

Four-week-old specific pathogen free male BALB/c mice were purchased from GemPharmatech Co., Ltd. (China). All procedures performed in the study involving animals were in compliance with the ARRIVE guidelines. To establish a prostate cancer model *in vivo*, DU145 cells (1 × 10^7^) were subcutaneously inoculated into the mice (shCtrl group). Further, to investigate the protective effect of TMED3 inhibition on prostate cancer metastasis, the mice were subcutaneously injected with shTMED3 (shTMED3 group) on day 10 after DU145 cell inoculation. Next, tumor tissues were collected on day 23 after DU145 cell inoculation. Hematoxylin and eosin (HE) staining was performed to observe morphological alterations in prostate tumors, and the IVIS Spectrum animal imaging system (LB983, Berthold Technologies, Belgium) was used to evaluate tumor growth and whole metastasis conditions after administering 100 μL of XenoLight D-luciferin Potassium Salt (15 mg/mL, Perkin Elmer, USA) to each mouse on day 23. All procedures on the mice followed the guidelines for humane treatment by the People’s Hospital of Guangxi Zhuang Autonomous Region (Number: K-Y-LW-2022-04).

### CCK-8 assay

DU145 cells were seeded in 96-well plates and infected with a lentivirus at different time points. Afterward, 10 μL of the CCK-8 solution was supplemented to each well, followed by incubation for 1 h at 37°C. Absorbance was measured at 450 nm using a microplate reader.

### Flow cytometry (FCM)

To determine the cell cycle and apoptosis of DU145 cells, we examined the positive expression of Annexin V and PI in DU145 cells by FCM. Briefly, DU145 cells were incubated with 10% fetal bovine serum (FBS). Then, the cells were incubated with the PI/RNase solution or phycoerythrin-PI and fluorescein isothiocyanate (FITC)-Annexin V for 30 min at 4°C in the dark. The cell apoptosis (early, late, and total apoptosis) and cell cycle (G0/G1, S, and G2/M phase) of DU145 cells were examined using a flow cytometer (Beckman Coulter, USA). PI^−^Annexin V^+^ cells were defined as cells at the early stage of apoptosis, whereas PI^+^Annexin V^+^ cells were defined as cells at the late stage of apoptosis.

### Migration and wound healing assays

After lentivirus infection, DU145 cells were seeded into the Transwell plates (upper chamber), and FBS was added to the lower chamber as a chemoattractant. After 24 h, the invading cells were stained with the 0.1% crystal violet staining solution and observed under a light microscope (Olympus, Tokyo, Japan). Cells infected with shTMED3 or shCtrl were scratched, wound healing was monitored, and the migration distance was imaged 24 h after scratch treatment.

### Western blotting

Prostate tumors and cells were homogenized for isolating total proteins, which were separated by sodium dodecyl sulfate–polyacrylamide gel electrophoresis and transferred to membranes. After blocking, the membranes were stained with anti-TMED3 (1:2000), anti-p-FOXO1a (1:2000), anti-FOXO1a (1:2000), anti-pFOXO3a (1:2000), anti-FOXO3a (1:2000), anti-laminB (1:2000), and anti-GAPDH (1:2000) antibodies, followed by staining with a secondary antibody (1:5000) and enhanced ECL detection kit.

### Real-time quantitative reverse transcriptase polymerase chain reaction (qRT-PCR)

Total RNA was extracted from the cells using the TRIzol reagent, and the extracted RNA was used to synthesize cDNA using the Promega M-MLV kit. qRT-PCR was performed using Light Cycler 480 SYBR Green I Master Mix, as described previously [20]. Primers used for qRT-PCR are listed in Suppl. Table 1.

### HE staining

The tumor tissues were harvested and fixed using 4% neutral buffered formalin before stepwise dehydration and embedded in paraffin. Then, the samples were stained with HE to observe morphological alterations under a light microscope (Olympus, Tokyo, Japan).

### Immunohistochemistry

The tumor tissue sections were deparaffinized and incubated in mixed phosphate-buffered saline containing 0.1% Triton X-100 and 3% H_2_O_2_ in the dark. After performing antigen retrieval, the sections were blocked with 5% normal goat serum and stained with anti-ki67 at 4°C overnight. The sections were incubated with the correspondent horseradish peroxidase-conjugated secondary antibodies, followed by DAB reactions and HE staining. Cells with brownish-yellow particles were considered ki67-positive cells.

### Immunofluorescence

For immunofluorescence staining, the slides were blocked, and anti-p-FOXO1a or anti-p-FOXO3a antibodies were directly added to the samples, followed by overnight incubation. Next, the samples were incubated with FITC-conjugated secondary antibodies. Finally, the slides were observed under a fluorescence microscope after nucleus staining.

### Statistical analysis

The data are presented as the mean ± standard deviation. *t*-test and one-way analysis of variance were performed to statistically analyze the data. Prostate cancer gene expression data were obtained from The Cancer Genome Atlas (TCGA) database. Visualization and Integrated Discovery were used to perform KEGG pathway enrichment analysis. *p* < 0.05 was considered statistically significant.

## Results

### Knockdown of TMED3 suppressed proliferation and promoted apoptosis in prostate cancer cells

**Figure 1:**
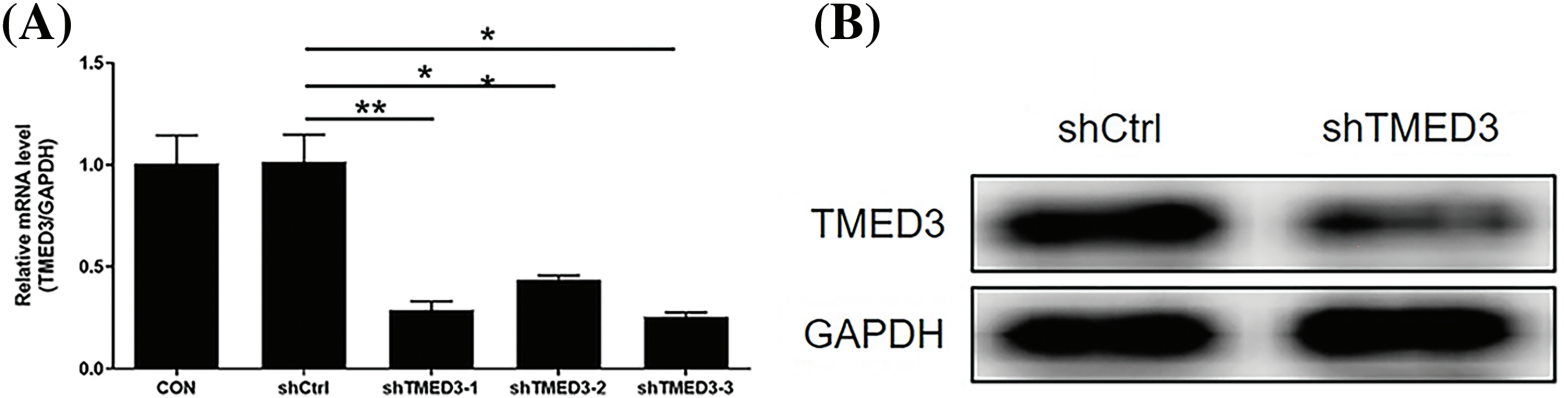
The silencing efficiency of shTMED3. (A) DU145 cells were treated with shTMED3-1, shTMED3-2, and shTMED3-3 for 72 h and subjected to qRT-PCR. Relative TMED3 mRNA expression in DU145 was analyzed. (B) DU145 cells were treated with shTMED3-3 for 72 h and subjected to western blotting. The TMED3 protein level in DU145 was determined. *p < 0.05; **p < 0.01.

The silencing efficiency of shTMED3-3 was the most potent among the three shTMED3s (Fig. 1A). The western blotting results showed that the levels of the TMED3 protein decreased significantly after shTMED3-3 intervention (Fig. 1B). Hence, we selected shTMED3-3 to inhibit TMED3 in DU145 cells in subsequent *in vitro* experiments.

**Figure 2:**
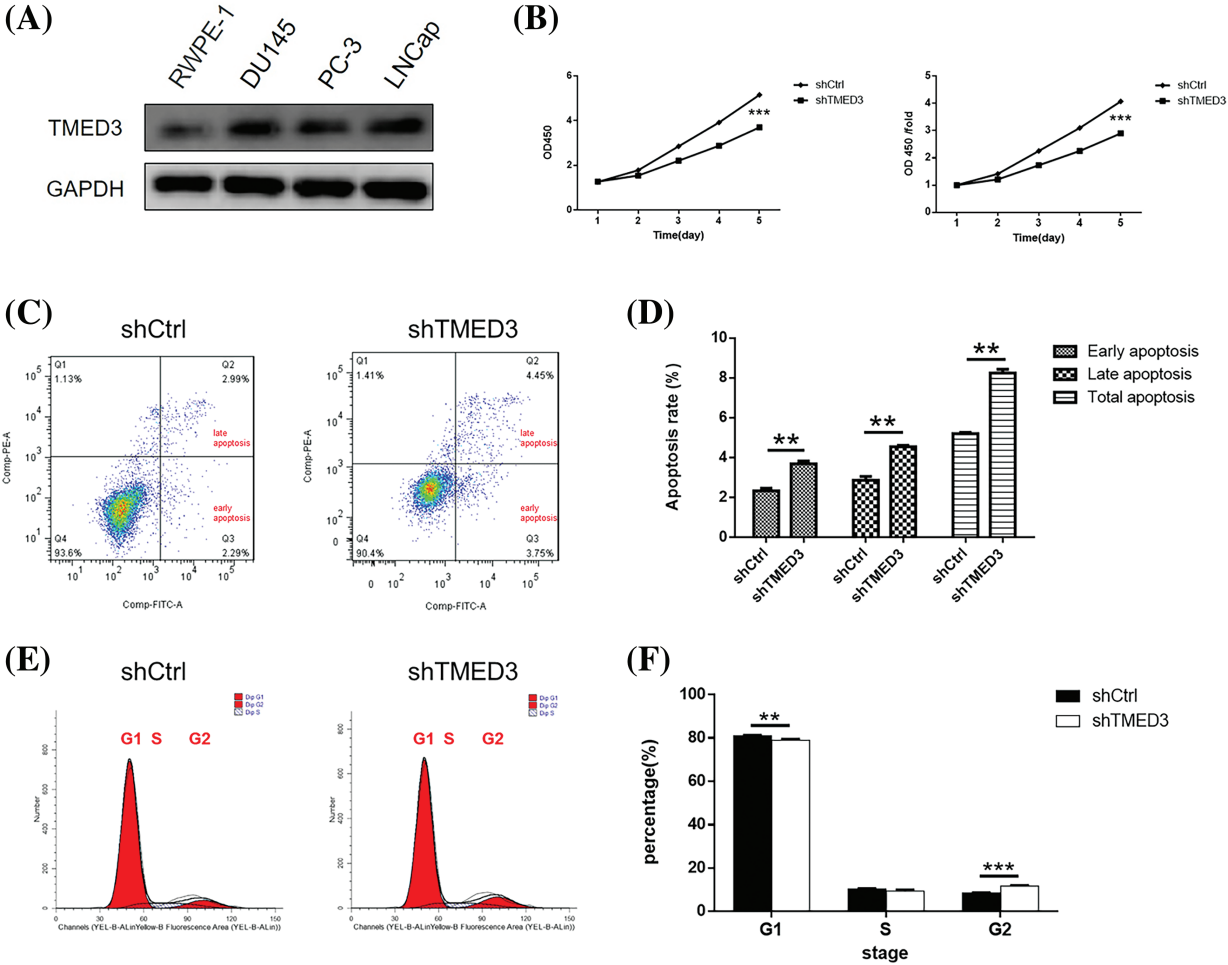
TMED3 regulated proliferation and promoted apoptosis in DU145 cells. (A) TMED3 protein levels in normal human prostate cells and prostate cancer cells were determined by western blotting. DU145 cells were infected with a lentivirus to silence TMED3 expression, and shCtrl was used as a scrambled control. (B) Cytotoxicity at different time points was determined by performing the CCK-8 assay. (C) A representative scatter plot of PI^+^Annexin V^+^ and PI^−^Annexin V^+^ DU145 cells by flow cytometry. (D) The proportions of DU145 cells at different stages of apoptosis (early apoptosis, late apoptosis, and total apoptosis) by flow cytometry. (E and F) The cell cycle (G1, S, and G2 phases) of DU145 cells was determined by flow cytometry. **p < 0.01; ***p < 0.001.

First, we observed the levels of the TMED3 protein in normal human prostate cells (RWPE-1 cells) and prostate cancer cells (DU145, PC-3, and LNCap cells). The results showed that TMED3 was more highly expressed in PC-3, LNCap, and DU145 cells than in RWPE-1 cells (Fig. 2A). Thus, the results indicated that TMED3 may play a crucial role in prostate cancer progression.

CCK-8 and FCM assays were performed to investigate the effect of TMED3 inhibition on prostate cancer cell growth. shTMED3 reduced cell growth in a time-dependent manner, and it decreased markedly on day 5 after shTMED3 intervention compared with that in the shCtrl group (Fig. 2B). The FCM results showed that the proportions of PI^+^Annexin V^+^ and PI^−^Annexin V^+^ cells in the shTMED3 group were higher than those in the shCtrl group (Figs. 2C and 2D). Additionally, TMED3 knockdown decreased the cell percentage at the G1 phase (Figs. 2E and 2F). These results indicated that TMED3 promoted cell proliferation and inhibited apoptosis in prostate cancer.

### Knockdown of TMED3 inhibited the invasion and migration of prostate cancer cells

**Figure 3:**
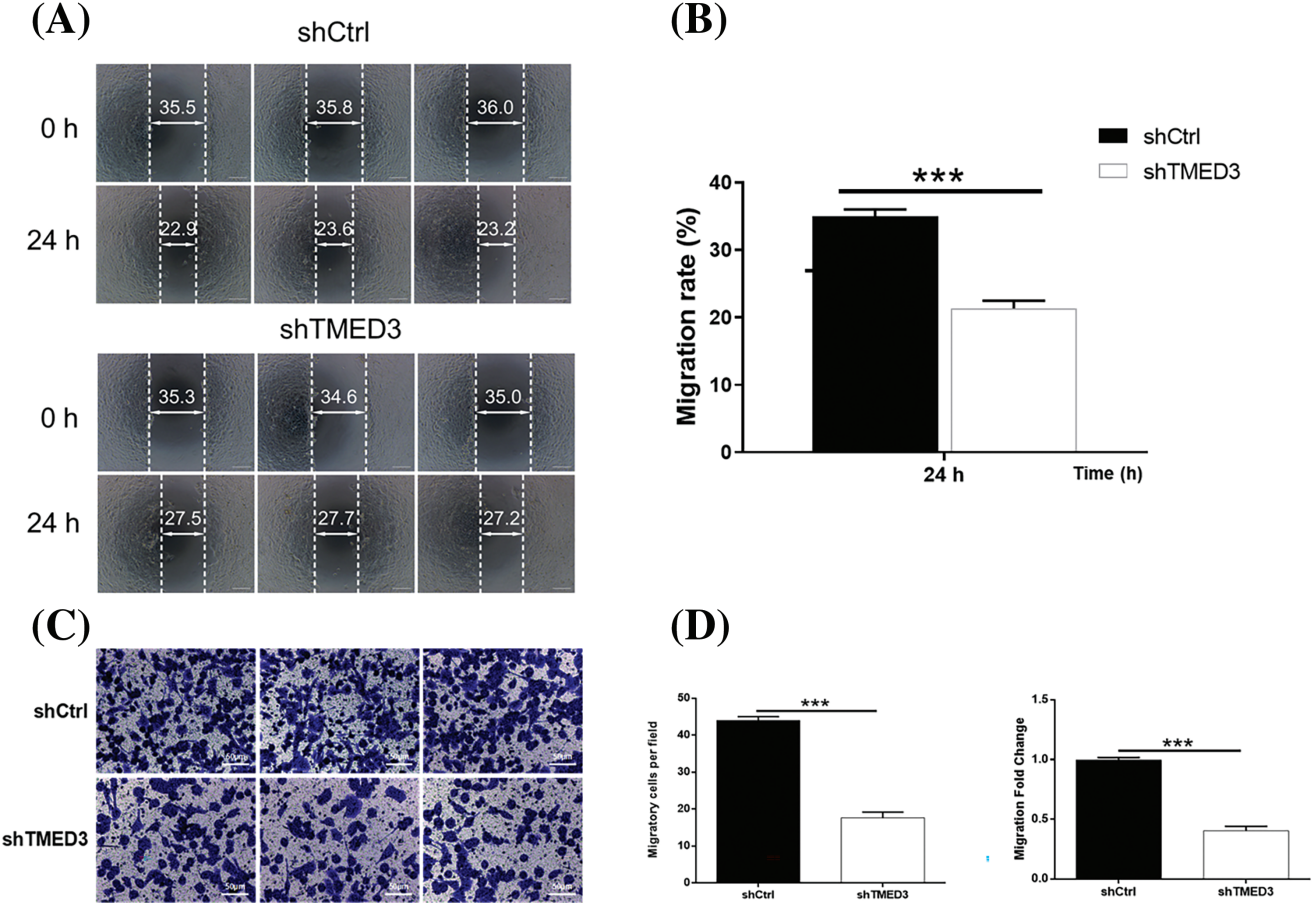
TMED3 regulated the invasion and migration of prostate cancer cells. DU145 cells were infected with a lentivirus to silence TMED3 expression, and shCtrl was used as a scrambled control. (A and B) The wound-healing assay was performed to determine the invasion ability of DU145 cells. (C and D) DU145 cell migration was assessed by performing the Transwell assay. ***p < 0.001. Original magnification, 200×.

We investigated alterations in the invasion and migration of prostate cancer cells induced by shTMED3. The invasion capacity of prostate cancer cells was suppressed by shTMED3 (Figs. 3A and 3B). Moreover, prostate cancer cells transfected with shTMED3 exhibited lower migration activity than did the control cells (Figs. 3C and 3D). Collectively, these results suggested that TMED3 knockdown could be an important defense mechanism against prostate cancer development.

### The correlation of the FOXO signaling pathway and TMED3 during the progression of prostate cancer

**Figure 4:**
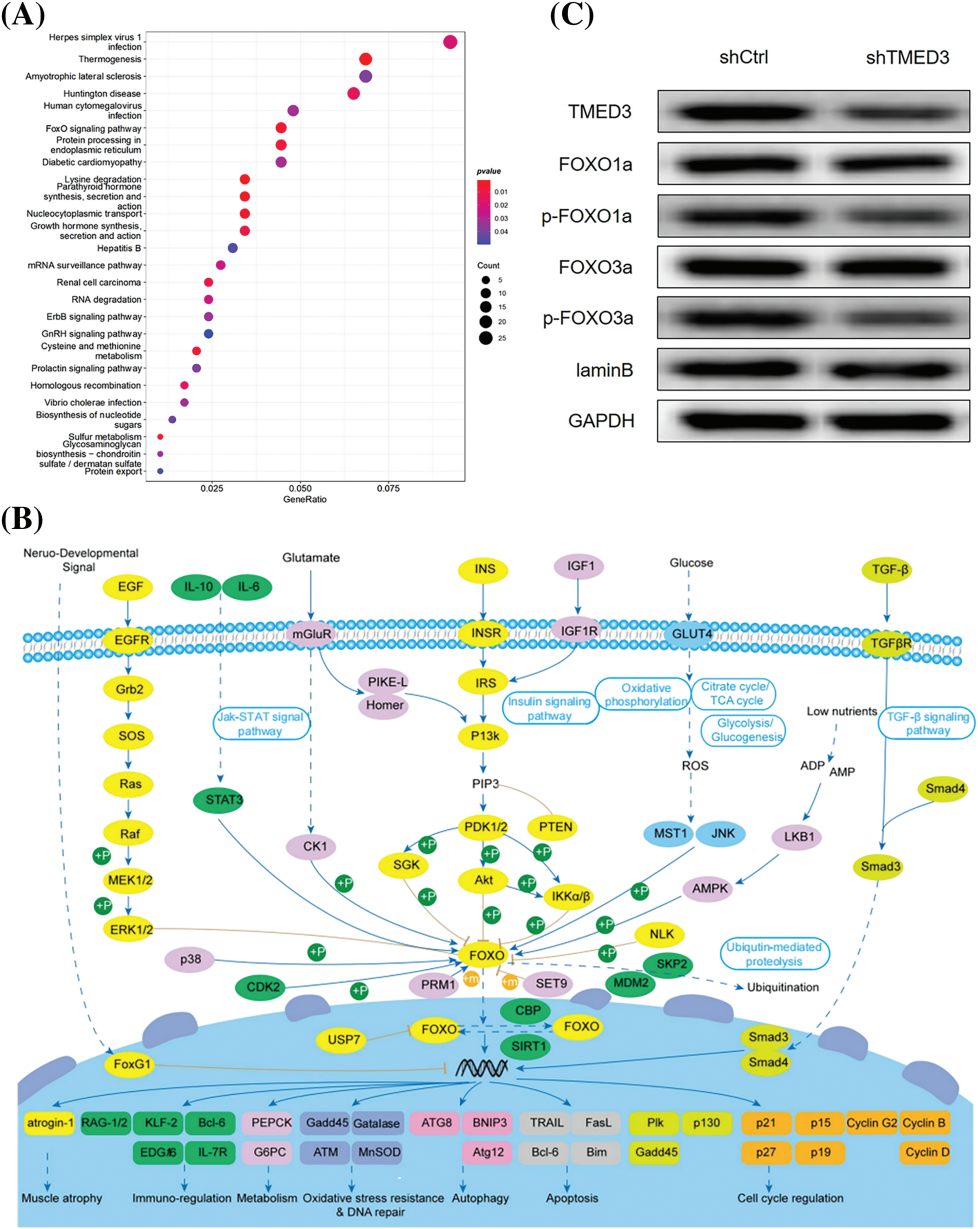
TMED3 inhibited the FOXO pathway by inducing FOXO1a and FOXO3a phosphorylation during prostate cancer progression. (A) The enrichment analysis of the TCGA data was performed to analyze the correlation of TMED3 with 26 signaling pathways. The sizes of the circles indicate the significance of enrichment, whereas their colors represent the strength and direction of the enrichment (red: positive correlation, blue: negative correlation). (B) The key role of the FOXO family in cancer-related pathways. (C) Western blotting of FOXO1a, p-FOXO1a, FOXO3a, and p-FOXO3a in DU145 cells.

To investigate the potential molecular mechanism underlying the role of TMED3 in prostate cancer, we used the TCGA database. We identified 26 signaling pathways that were correlated to TMED3 mRNA levels. Among them, the FOXO pathway plays a protective role in prostate cancer, and a strong correlation was observed between TMED3 and the FOXO pathway (Figs. 4A and 4B). Thus, we focused on the potential cascade correlation between TMED3 and the FOXO pathway.

To better determine whether the marked TMED3 upregulation was a driving cause for FOXO signaling pathway activation or a correlative hallmark during prostate cancer progression, TMED3 was downregulated by injecting shTMED3 *in vivo*. TMED3 protein levels in the tumor tissues decreased significantly after shTMED3 administration *in vivo*. Furthermore, TMED3 silencing decreased FOXO1a and FOXO3a phosphorylation (Fig. 4C). Thus, the results indicated that TMED3 inhibited the FOXO pathway by inducing FOXO1a and FOXO3a phosphorylation during prostate cancer progression.

### TMED3 downregulation suppressed prostate cancer progression by decreasing FOXO1a and FOXO3a phosphorylation in vivo

**Figure 5:**
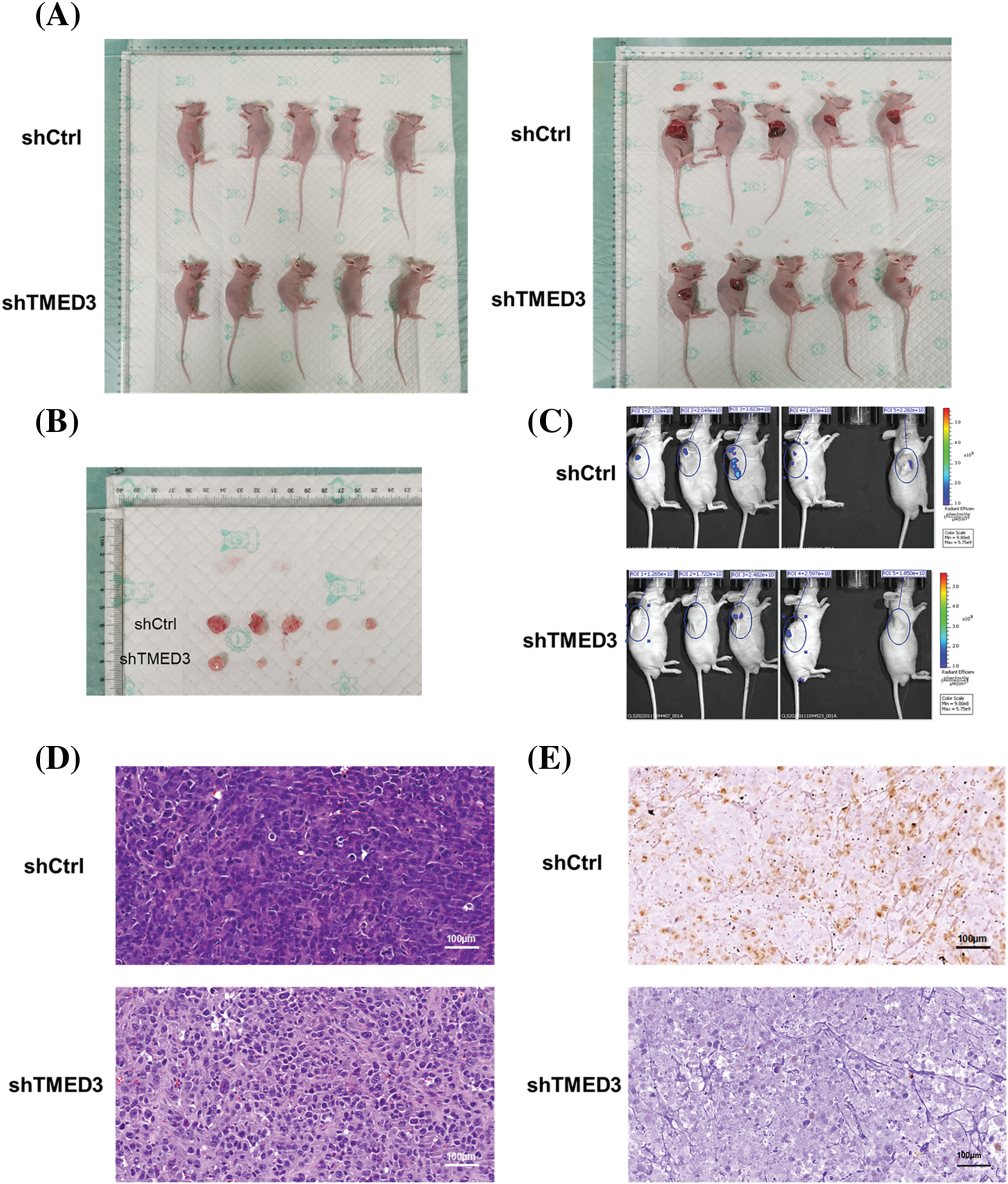
TMED3 downregulation alleviated prostate cancer *in vivo*. (A and B) The sizes of prostate tumors in mice. (C) Prostate cancer metastasis in mice was evaluated using an *in vivo* imaging system. (D) Histological alterations in prostate tumors were observed by performing HE staining. (E) Immunohistochemistry of ki67-positive expression in prostate tumors of mice. Original magnification, 400×.

**Figure 6:**
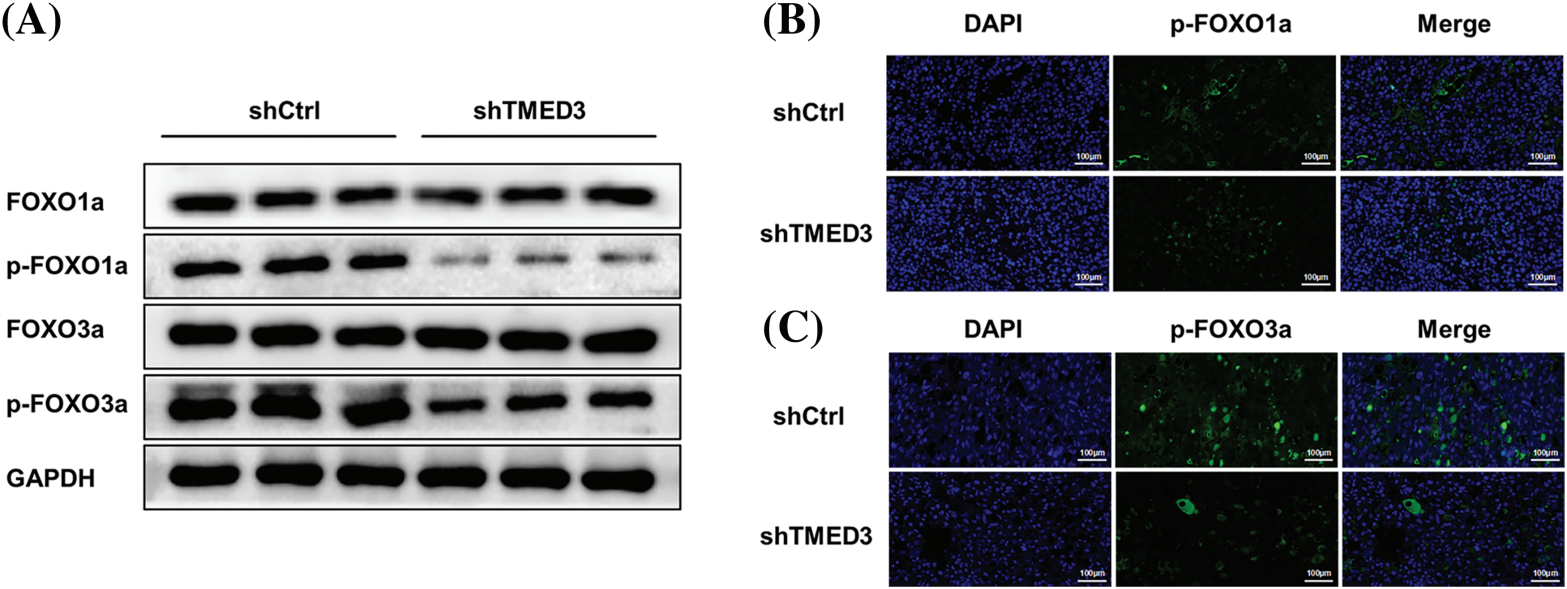
TMED3 downregulation decreased p-FOXO1 and p-FOXO3 levels in a mouse model of prostate cancer. (A) Western blotting of FOXO1a, p-FOXO1a, FOXO3a, and p-FOXO3a in tumor tissues. Immunofluorescence analysis of p-FOXO1a (B) and p-FOXO3a (C) in tumor tissues. Original magnification, 200×.

We investigated whether TMED3 downregulation alleviated prostate cancer in mice. TMED3 downregulation greatly inhibited prostate tumor growth in the mice (Figs. 5A and 5B). Next, we observed prostate cancer cell metastasis using an *in vivo* imaging system. Mice in the shCtrl group showed prostate cancer metastasis (Fig. 5C). However, compared with mice injected with shCtrl cells, mice injected with TMED3-downregulated prostate cancer cells showed lower prostate cancer metastasis *in vivo* on day 23 after the treatment. The histological analysis indicated that mice receiving shTMED3 showed a lower number of prostate cancer foci than did the mice in the shCtrl group (Fig. 5D). The IHC results showed that TMED3-downregulated mice exhibited a lower percentage of Ki67^+^ tumor cells than did mice in the shCtrl group, which implied that TMED3 was associated with the modulatory process of apoptosis in prostate cancer (Fig. 5E). Based on this mouse model of prostate cancer, we confirmed that TMED3 downregulation decreased p-FOXO1a and p-FOXO3a levels rather than FOXO1a and FOXO3a levels (Fig. 6). These findings support the notion that the TMED3-regulated phosphorylation of FOXO1a and FOXO3a was involved in prostate cancer progression.

## Discussion

In this study, we found that TMED3 protein levels increased in prostate cancer cells, and TMED3 knockdown inhibited the proliferation, migration, and invasion and promoted the apoptosis of prostate cancer cells *in vitro*. The KEGG pathway enrichment analysis revealed a strong correlation between TMED3 and FOXO signaling pathway phosphorylation. Additional experiments confirmed that the TMED3-induced phosphorylation of FOXO1a and FOXO3a played a role in prostate cancer progression. The *in vivo* experiments showed that TMED3 downregulation suppressed prostate cancer progression in mice. These findings provide novel evidence that TMED3 downregulation is a promising strategy to counteract tumor cell proliferation, migration, and invasion.

Accumulating evidence shows that TMED3 is a newly identified cancer-related protein in several malignancies, such as breast cancer, gastric cancer, chordoma, and lung cancer. TMED3 exerts antitumor effects by downregulating Wnt/β-catenin signaling via AKTO regulation [21]. Zhang et al. reported that TMED3 enhanced cell viability and migration and inhibited apoptosis in chordoma cells by greatly silencing TMED3 expression and activation, suggesting that TMED3 may be a novel target for tumor therapy [11]. Guo et al. found that TMED3 protein levels were high in malignant melanoma; thus, they identified TMED3 as a crucial tumor-promoting gene in malignant melanoma progression [22]. However, evidence on the role of TMED3 in prostate cancer is lacking. Herein, we compared TMED3 protein levels between normal human prostate cells and prostate cancer cells and found its higher levels in prostate cancer cells than in normal cells. Tissue homeostasis is maintained through a balance of proliferative and antiproliferative signals, and tumor cell migration and invasion are considered key indicators to evaluate the capacity of tumor metastasis [23–27]. Herein, we silenced TMED3 expression both *in vitro* and *in vivo*. The *in vitro* results showed that TMED3 knockdown in prostate cancer cells inhibited the proliferation, migration, and invasion and promoted the apoptosis of prostate cancer cells. The *in vivo* results showed that TMED3 downregulation suppressed the apoptosis of prostate tumor cells and alleviated prostate cancer. The present findings imply that TMED3 is a novel inducer of prostate cancer progression, and its downregulation may hold prognostic value for metastasis.

TMED3 is involved in multiple pathways that may contribute to prostate cancer development [8,28,29]. Herein, the KEGG pathway enrichment analysis was performed to investigate the possible downstream cooperator of TMED3 in prostate cancer, and a strong correlation between TMED3 and FOXO signaling pathway phosphorylation was observed. FOXO is an important downstream effector and transcriptional regulator associated with tumor metastasis [30–32]. Growing evidence shows that FOXO proteins, including FOXO1a and FOXO3a, suppress tumors via being regulated by oncogenic proteins such as Akt, inhibitor of nuclear factor-κB kinase, and S-phase kinase-associated protein 2 [33–35]. Boudjadi et al. demonstrated that fibroblast growth factor 8, a direct transcriptional target of paired box gene 3 (PAX3)–FOXO1, was responsible for PAX3–FOXO1-independent tumor recurrence via an autocrine mechanism, implying the crucial role of FOXO1 in tumor metastasis [36]. Liu et al. reported that FOXO1 phosphorylation regulated by cyclin-dependent kinase 1 played a role in tumorigenesis by promoting cell proliferation and survival [16]. Additionally, FOXO3a modulated Wnt/β-catenin signaling and suppressed EMT in prostate cancer cells [18]. Therefore, we hypothesized that the TMED3-induced phosphorylation of FOXO1a and FOXO3a may regulate the proliferation, migration, invasion, and apoptosis of prostate cancer cells. Herein, we confirmed that FOXO1a and FOXO3a phosphorylation was regulated by TMED3. High TMED3 expression resulted in the loss of function of FOXO1a and FOXO3a owing to S249 phosphorylation, thereby promoting prostate cancer development.

Nevertheless, this study has several limitations that need further investigation. The reason behind TMED3 activation in prostate cancer was not clarified. Thus, future studies are warranted to investigate whether FOXO signaling pathway activation alleviates prostate cancer.

## Conclusion

We demonstrated that TMED3 phosphorylated FOXO1a and FOXO3a, thereby contributing to the loss of their transcriptional activities. TMED3 inhibition suppressed prostate cancer cell metastasis *in vivo* and *in vitro*. The present findings suggested that TMED3 inhibition may serve as a potent therapeutic target against prostate cancer progression.

## Supplementary Materials

**Table 1:** Gene-specific primers

Gene	Forward primer	Reverse primer
TMED3	TGAAGTCAAGGGCGTTTAT	TGTCTGGGAGAATGGGAG
GAPDH	TGACTTCAACAGCGACACCCA	CACCCTGTTGCTGTAGCCAAA

## Data Availability

The data that support the findings of this study are available on request from the corresponding author upon reasonable request.

## References

[1] Culig, Z., Santer, F. R. (2014). Androgen receptor signaling in prostate cancer. Cancer Metastasis Reviews*,* 33 *(*2–3*),* 413–427. 10.1007/s10555-013-9474-0; 24384911

[2] Green, S. M., Mostaghel, E. A., Nelson, P. S. (2012). Androgen action and metabolism in prostate cancer. Molecular and Cellular Endocrinology*,* 360 *(*1–2*),* 3–13. 10.1016/j.mce.2011.09.046; 22453214 PMC4124858

[3] Nguyen-Nielsen, M., Borre, M. (2016). Diagnostic and therapeutic strategies for prostate cancer. Seminars in Nuclear Medicine*,* 46 *(*6*),* 484–490. 10.1053/j.semnuclmed.2016.07.002; 27825428

[4] Zhou, L., Song, Z., Hu, J., Liu, L., Hou, Y. et al. (2021). ACSS3 represses prostate cancer progression through downregulating lipid droplet-associated protein PLIN3. Theranostics*,* 11 *(*2*),* 841–860. 10.7150/thno.49384; 33391508 PMC7738848

[5] Gupta, P., Serajuddin, M. (2021). Fish lipid against prostate cancer (PC-3) through apoptosis and cell cycle arrest. Nutrition and Cancer*,* 73 *(*2*),* 300–306. 10.1080/01635581.2020.1743872; 32242459

[6] Shen, Z., Zhou, L., Zhang, C., Xu, J. (2020). Reduction of circular RNA Foxo3 promotes prostate cancer progression and chemoresistance to docetaxel. Cancer Letters*,* 468*,* 88–101. 10.1016/j.canlet.2019.10.006; 31593800

[7] Sun, L., Lü, J., Ding, S., Bi, D., Ding, K. et al. (2017). HCRP1 regulates proliferation, invasion, and drug resistance via EGFR signaling in prostate cancer. Biomedicine & Pharmacotherapy*,* 91*,* 202–207. 10.1016/j.biopha.2017.04.040; 28458158

[8] Duquet, A., Melotti, A., Mishra, S., Malerba, M., Seth, C. et al. (2014). A novel genome-wide in vivo screen for metastatic suppressors in human colon cancer identifies the positive WNT-TCF pathway modulators TMED3 and SOX12. EMBO Molecular Medicine*,* 6 *(*7*),* 882–901. 10.15252/emmm.201303799; 24920608 PMC4119353

[9] Jenne, N., Frey, K., Brugger, B., Wieland, F. T. (2002). Oligomeric state and stoichiometry of p24 proteins in the early secretory pathway. The Journal of Biological Chemistry*,* 277 *(*48*),* 46504–46511. 10.1074/jbc.M206989200; 12237308

[10] Strating, J. R., Martens, GJ. (2009). The p24 family and selective transport processes at the ER-Golgi interface. Biology of the Cell*,* 101 *(*9*),* 495–509. 10.1042/BC20080233; 19566487

[11] Zhang, X., Luo, Y., Li, Q. (2020). TMED3 promotes proliferation and migration in breast cancer cells by activating Wnt/β-catenin signaling. OncoTargets and Therapy*,* 13*,* 5819–5830. 10.2147/OTT.S250766; 32606792 PMC7311187

[12] Zheng, H., Yang, Y., Han, J., Jiang, W. H., Chen, C. et al. (2016). TMED3 promotes hepatocellular carcinoma progression via IL-11/STAT3 signaling. Scientific Reports*,* 6*,* 37070. 10.1038/srep37070; 27901021 PMC5128793

[13] Vainio, P., Mpindi, J. P., Kohonen, P., Fey, V., Mirtti, T. et al. (2012). High-throughput transcriptomic and RNAi analysis identifies AIM1, ERGIC1, TMED3 and TPX2 as potential drug targets in prostate cancer. PLoS One*,* 7 *(*6*),* e39801. 10.1371/journal.pone.0039801; 22761906 PMC3386189

[14] Khor, Y. S., Wong, P. F. (2024). MicroRNAs-associated with FOXO3 in cellular senescence and other stress responses. Biogerontology*,* 25 *(*1*),* 23–51. 10.1007/s10522-023-10059-6; 37646881

[15] Vyavahare, S., Kumar, S., Smith, K., Mendhe, B., Zhong, R. et al. (2023). Inhibiting MicroRNA-141-3p improves musculoskeletal health in aged mice. Aging and Disease*,* 14 *(*6*),* 2303–2316. 10.14336/AD.2023.0310-1; 37199586 PMC10676793

[16] Liu, P., Kao, T. P., Huang, H. (2008). CDK1 promotes cell proliferation and survival via phosphorylation and inhibition of FOXO1 transcription factor. Oncogene*,* 27 *(*34*),* 4733–4744. 10.1038/onc.2008.104; 18408765

[17] Shukla, S., Bhaskaran, N., Maclennan, G. T., Gupta, S. (2013). Deregulation of FoxO3a accelerates prostate cancer progression in TRAMP mice. Prostate*,* 73 *(*14*),* 1507–1517. 10.1002/pros.22698; 23765843 PMC4018753

[18] Liu, H., Yin, J., Wang, H., Jiang, G., Deng, M. et al. (2015). FOXO3a modulates WNT/β-catenin signaling and suppresses epithelial-to-mesenchymal transition in prostate cancer cells. Cellular Signalling*,* 27 *(*3*),* 510–518. 10.1016/j.cellsig.2015.01.001; 25578861

[19] Kong, Z., Deng, T., Zhang, M., Zhao, Z., Liu, Y. et al. (2018). β-arrestin1-medieated inhibition of FOXO3a contributes to prostate cancer cell growth *in vitro* and *in vivo*. Cancer Science*,* 109 *(*6*),* 1834–1842. 10.1111/cas.13619; 29676828 PMC5989847

[20] Zhang, X., Hu, X., Zhang, Y., Liu, B., Pan, H. et al. (2023). Impaired autophagy-accelerated senescence of alveolar type II epithelial cells drives pulmonary fibrosis induced by single-walled carbon nanotubes. Journal of Nanobiotechnology*,* 21 *(*1*),* 69. 10.1186/s12951-023-01821-6; 36849924 PMC9970859

[21] Zhang, D., Sun, L., Zhang, J. (2021). TMED3 exerts a protumor function in non-small cell lung cancer by enhancing the Wnt/β-catenin pathway via regulation of AKT. Toxicology and Applied Pharmacology*,* 433*,* 115793. 10.1016/j.taap.2021.115793; 34758370

[22] Guo, X., Yin, X., Xu, Y., Li, L., Yuan, M. et al. (2023). TMED3 promotes the development of malignant melanoma by targeting CDCA8 and regulating PI3K/Akt pathway. Cell & Bioscience*,* 13 *(*1*),* 65. 10.1186/s13578-023-01006-6; 36991473 PMC10053972

[23] Dai, X., Liang, Z., Liu, L., Guo, K., Xu, S. et al. (2019). Silencing of MALAT1 inhibits migration and invasion by sponging miR-1-3p in prostate cancer cells. Molecular Medicine Reports*,* 20 *(*4*),* 3499–3508. 10.3892/mmr.2019.10602; 31485645 PMC6755148

[24] Eerola, S. K., Santio, N. M., Rinne, S., Kouvonen, P., Corthals, G. L. et al. (2019). Phosphorylation of NFATC1 at PIM1 target sites is essential for its ability to promote prostate cancer cell migration and invasion. Cell Communication and Signaling*,* 17 *(*1*),* 148. 10.1186/s12964-019-0463-y; 31730483 PMC6858710

[25] Jiang, C., Zhang, N., Hu, X., Wang, H. (2021). Tumor-associated exosomes promote lung cancer metastasis through multiple mechanisms. Molecular Cancer*,* 20 *(*1*),* 117. 10.1186/s12943-021-01411-w; 34511114 PMC8436438

[26] Ma, L., He, H., Jiang, K., Jiang, P., He, H. et al. (2020). FAM46C inhibits cell proliferation and cell cycle progression and promotes apoptosis through PTEN/AKT signaling pathway and is associated with chemosensitivity in prostate cancer. Sedentary Life and Nutrition*,* 12 *(*7*),* 6352–6369. 10.18632/aging.103030; 32283544 PMC7185131

[27] Townson, J. L., Naumov, G. N., Chambers, A. F. (2003). The role of apoptosis in tumor progression and metastasis. Current Molecular Medicine*,* 3 *(*7*),* 631–642. 10.2174/1566524033479483; 14601637

[28] Pei, J., Zhang, S., Yang, X., Han, C., Pan, Y. et al. (2021). Long non-coding RNA RP11-283G6.5 confines breast cancer development through modulating miR-188-3p/TMED3/Wnt/β-catenin signalling. RNA Biology*,* 18 *(*sup1*),* 287–302. 10.1080/15476286.2021.1941608; 34130584 PMC8677045

[29] Shorning, B. Y., Dass, M. S., Smalley, M. J., Pearson, H. B. (2020). The PI3K-AKT-mTOR pathway and prostate cancer: At the crossroads of AR, MAPK, and WNT signaling. International Journal of Molecular Sciences*,* 21 *(*12*),* 4507. 10.3390/ijms21124507; 32630372 PMC7350257

[30] Du, W. W., Fang, L., Yang, W., Wu, N., Awan, F. M. et al. (2017). Induction of tumor apoptosis through a circular RNA enhancing Foxo3 activity. Cell Death and Differentiation*,* 24 *(*2*),* 357–370. 10.1038/cdd.2016.133; 27886165 PMC5299715

[31] Wang, Y., Lyu, Z., Qin, Y., Wang, X., Sun, L. et al. (2020). FOXO1 promotes tumor progression by increased M2 macrophage infiltration in esophageal squamous cell carcinoma. Theranostics*,* 10 *(*25*),* 11535–11548. 10.7150/thno.45261; 33052231 PMC7546008

[32] Watkins, S. K., Zhu, Z., Riboldi, E., Shafer-Weaver, K. A., Stagliano, K. E. et al. (2011). FOXO3 programs tumor-associated DCs to become tolerogenic in human and murine prostate cancer. The Journal of Clinical Investigation*,* 121 *(*4*),* 1361–1372. 10.1172/JCI44325; 21436588 PMC3069771

[33] Brunet, A., Bonni, A., Zigmond, M. J., Lin, M. Z., Juo, P. et al. (1999). Akt promotes cell survival by phosphorylating and inhibiting a Forkhead transcription factor. Cell*,* 96 *(*6*),* 857–868. 10.1016/s0092-8674(00)80595-4; 10102273

[34] Hu, M. C., Lee, D. F., Xia, W., Golfman, L. S., Ou-Yang, F. et al. (2004). IkappaB kinase promotes tumorigenesis through inhibition of forkhead FOXO3a. Cell*,* 117 *(*2*),* 225–237. 10.1016/s0092-8674(04)00302-2; 15084260

[35] Huang, H., Cheville, J. C., Pan, Y., Roche, P. C., Schmidt, L. J. et al. (2001). PTEN induces chemosensitivity in PTEN-mutated prostate cancer cells by suppression of Bcl-2 expression. The Journal of Biological Chemistry*,* 276 *(*42*),* 38830–38836. 10.1074/jbc.M103632200; 11495901

[36] Boudjadi, S., Pandey, P. R., Chatterjee, B., Nguyen, T. H., Sun, W. et al. (2021). A fusion transcription factor-driven cancer progresses to a fusion-independent relapse via constitutive activation of a downstream transcriptional target. Cancer Research*,* 81 *(*11*),* 2930–2942. 10.1158/0008-5472.CAN-20-1613; 33589519 PMC8178207

